# Drug use, HIV, HCV and TB: major interlinked challenges in Eastern Europe and Central Asia

**DOI:** 10.7448/IAS.17.4.19501

**Published:** 2014-11-02

**Authors:** Michel Kazatchkine

**Affiliations:** United Nations Special Envoy for HIV/AIDS in Eastern Europe and Central Asia, Geneva, Switzerland

## Abstract

Eastern Europe and Central Asia have the largest drug epidemic globally and the fastest and still expanding HIV epidemic. The Russian Federation and Ukraine together account for over 90% of the reported AIDS cases in the region. If small in absolute numbers, the epidemics are however significant in prevalence rate in most countries of Central Asia. Most heroin and many of the new synthetic or home-made drugs are injected, which has led to high prevalence levels (up to 90%) of HCV infection in people who inject drugs (PWID). The two epidemics of HIV and HCV are in turn interlinked with TB and MDR-TB that are highly prevalent among marginalized populations in the region. Despite progress in the last two years, access to antiretroviral treatment remains far below global levels and increases more slowly than new reported cases of HIV. Access to prevention is limited with low coverage of needle exchange programs and very low or inexistent access to opioid substitutive therapy. There are few exceptions to this situation, including Ukraine where harm reduction programs are being scaled up together with significant peer outreach programs for PWIDs. This is likely to be the reason why the epidemic curves in the Russian Federation and Ukraine are now diverging. The region faces many structural, cultural, societal and political obstacles in responding to these quadruple epidemics. Without a significantly expanded and strengthened response, these epidemics will remain major causes of illness and premature deaths in the region.

